# Intelligent Control of Bulk Tobacco Curing Schedule Using LS-SVM- and ANFIS-Based Multi-Sensor Data Fusion Approaches

**DOI:** 10.3390/s19081778

**Published:** 2019-04-13

**Authors:** Juan Wu, Simon X. Yang

**Affiliations:** 1 School of Microelectronics and Communication Engineering, Chongqing University, Chongqing 400044, China; juanwucq@126.com; 2 Chongqing College of Electronic Engineering, Chongqing 401331, China; 3 School of Engineering, University of Guelph, Guelph, ON N1G 2W1, Canada

**Keywords:** multi-sensor data fusion, electronic nose, bulk tobacco curing schedule, least squares support vector machines, adaptive neuro-fuzzy inference system

## Abstract

The bulk tobacco flue-curing process is followed by a bulk tobacco curing schedule, which is typically pre-set at the beginning and might be adjusted by the curer to accommodate the need for tobacco leaves during curing. In this study, the controlled parameters of a bulk tobacco curing schedule were presented, which is significant for the systematic modelling of an intelligent tobacco flue-curing process. To fully imitate the curer’s control of the bulk tobacco curing schedule, three types of sensors were applied, namely, a gas sensor, image sensor, and moisture sensor. Feature extraction methods were given forward to extract the odor, image, and moisture features of the tobacco leaves individually. Three multi-sensor data fusion schemes were applied, where a least squares support vector machines (LS-SVM) regression model and adaptive neuro-fuzzy inference system (ANFIS) decision model were used. Four experiments were conducted from July to September 2014, with a total of 603 measurement points, ensuring the results’ robustness and validness. The results demonstrate that a hybrid fusion scheme achieves a superior prediction performance with the coefficients of determination of the controlled parameters, reaching 0.9991, 0.9589, and 0.9479, respectively. The high prediction accuracy made the proposed hybrid fusion scheme a feasible, reliable, and effective method to intelligently control over the tobacco curing schedule.

## 1. Introduction

The tobacco drying process has a great influence on the quality of cigarette smoke [1,2]. The methods of tobacco drying are various all around the world, such as solar energy drying [3], hot water drying [1,4], and the flue-curing method. In China, the flue-curing method is mostly applied, which incorporates airflow and temperature control in bulk curing barns [5]. Specifically, temperature control refers to keeping the dry-bulb temperature (DBT) and wet-bulb temperature (WBT) of the curing barn consistent with the bulk tobacco curing schedule, which is designed based on the three-stage curing theory. In this theory, according to the changes in the chemical and physical characteristics of tobacco leaves, the bulk tobacco curing process could be divided into three stages, namely: leaf-yellowing, leaf-drying, and stem-drying. During curing, the curer, who is a professional worker, observes the tobacco curing condition so as to adjust the bulk curing schedule in order to make sure the fragrant tobacco leaves be cured out.

Previous research on the tobacco curing control process mainly focused on how to automatically implement temperature control in bulk curing barns. Fuzzy-based control methods have been mostly employed [4,6,7]. These methods keep the measured DBT or WBT in the curing barn consistent with the set DBT or WBT of the bulk curing schedule. It has been noted that if the measured DBT is greater than the set DBT, the heating apparatus of the curing barn will be turned off, otherwise it will be turned on. In the above work, how to set DBT and WBT of tobacco curing schedule is not discussed. The setpoint values of the DBT and WBT are the key parameters adjusted by the curer in the curing schedule. As though they are pre-set at the beginning of the flue-curing process, during curing, the curer still needs to adjust the setpoint values depending on the specific curing condition of the tobacco leaves in the curing barn. For example, if the tobacco leaves have almost turned to yellow while the setpoint value of the DBT still remains 42 °C, the curer might change it to 48 °C in order to ensure that the stems of the tobacco leaves will be cured to yellow. Therefore, it is necessary and meaningful to research how to intelligently set DBT and WBT in the curing schedule during the curing process. In one of the few studies that involved an intelligent bulk tobacco curing control, Zhang, Jiang, and Chen [8] proposed an intelligent tobacco curing control technique based on color recognition by means of the fuzzy logical method, but the approach to utilizing the color features to control the flue-curing process was not presented. Wu, Yang, and Tian [9]; Wang et al. [10]; and Wu, Yang, and Tian [11] applied the flue-curing control methods that intelligently set the DBT and WBT of the bulk tobacco curing schedule based on real-time image features, which could not fully reflect the characteristics changes of the tobacco leaves.

Aiming to achieve intelligent control, the curer’s operation for adjusting the curing schedule should be analyzed first. As the bulk tobacco curing schedule is pre-set beforehand, the tobacco leaves might not be cured properly if following this pre-set curing schedule [12]. In fact, the curer’s changes to the pre-set curing schedule to make it suit the actual curing condition, which involves adjusting the setpoint values of DBT and WBT, as well as the duration from current curing stage to the next. The most significant factor that will lead a curer to make adjustments in the curing schedule is the color of the tobacco leaves. Specifically, if the tobacco leaves have not turned to yellow at the end of the yellowing stage, the curer may increase the setpoint value of DBT or make the setpoint change to a longer time. To simulate this feature, a machine vision system was employed in this study. In addition, the chemical odor released by the tobacco leaves has important information for analyzing the curing process. In the traditional way, the curer might not smell the odor of the tobacco leaves for the toxicity of the odor during the curing process [13]. The chemicals emitted from the leaves during the curing process are harmful to the health of the curers. The airborne nicotine concentrations in the curing barns and the front yard of the curing barn were very high [14]. Many tobacco farmers had died from a variety of diseases after years of exposure to smoke, including lung or blood cancer, liver cirrhosis, or kidney failure [13]. An electronic nose (E-nose), which simulates the function of human’s nose, can solve this problem. It can “smell” what humans smell and what they are not able to [15,16,17,18]. E-noses have been widely used in tobacco-related research, such as for the suppression of background interference on odor data during tobacco curing [19], and for the identification of cigarette brands [20,21,22,23]. Consequently, this system added an E-nose in order to investigate the odor during tobacco’s curing. Furthermore, the moisture content of the tobacco leaves is also a key parameter to be considered, thus a moisture detector was included. Therefore, three types of sensors were applied in this study, that is, image, gas, and moisture sensors.

The next issue would be how to effectively make use of the multi-sensor data in order to achieve a good performance in the intelligent control process. Zhang et al. [24] applied the artificial neural network (ANN) method to process the odor, image, moisture, and other extracted features of the tobacco to achieve automatic control of the curing process. As a result of the high performance of the multi-sensor data fusion method on the noise elimination to the process control application [25], it was chosen in order to analyze the control process of a bulk tobacco curing schedule in this study. The multi-sensor data fusion method has been widely used in various research areas [17,26,27,28,29,30,31]. In the field of artificial sensors’ applications, which are highly related to the present study, a feature level fusion with principal component analysis (PCA) feature selection method and several pattern analysis techniques, such as ANN, linear discriminant analysis (LDA), partial least square (PLS), and support vector machine (SVM), have been mostly used for food authentication and the on-line monitoring of food fermentation processes [30,32,33,34]. These applications mostly employed either a feature level or decision level fusion method.

Inspired by the aforementioned applications of multi-sensor data fusion, we proposed three multi-sensor data fusion schemes to implement the intelligent control of a bulk tobacco curing schedule. The proposed fusion approach could make full considerations for the fused information of gas, image, and moisture sensors, so that a more accurate determination on the adjustments of the curing schedule could be achieved.

## 2. Materials and Methods

### 2.1. Materials and System Set-Up

**Figure 1:**
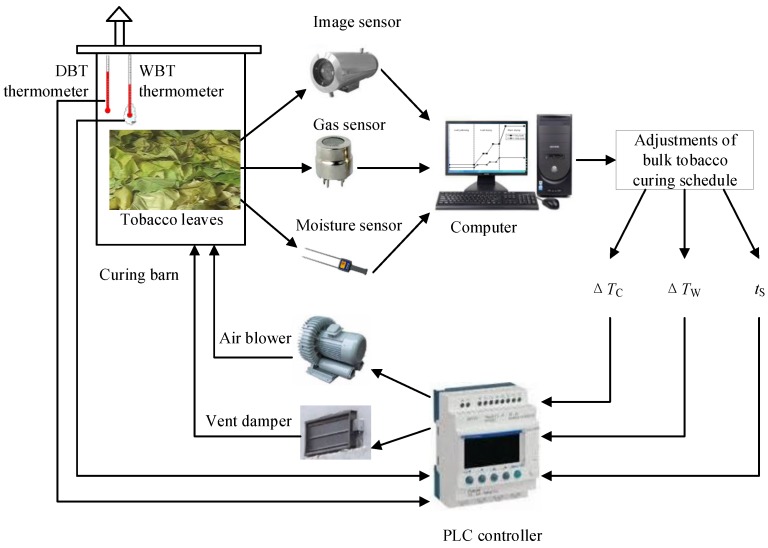
Framework of the proposed intelligent control method for bulk tobacco curing schedule. PLC—programmable logic controller; DBT—dry-bulb temperature; WBT—wet-bulb temperature.

A gas sensor, image sensor, and moisture sensor were employed to observe the curing condition of the tobacco leaves. The framework of the proposed intelligent control method for the tobacco curing schedule is shown in Figure 1. Three types of sensor data were collected, and all were sent to a computer to process and analyze. After computer processing the multi-sensor data and deciding how to adjust the bulk curing schedule, the adjustment decision was sent to the programmable logic controller (PLC), which received this order and controlled the air blower and vent damper in order to make sure the measured DBT and WBT of the curing barn would just follow this modified curing schedule. 

**Figure 2:**
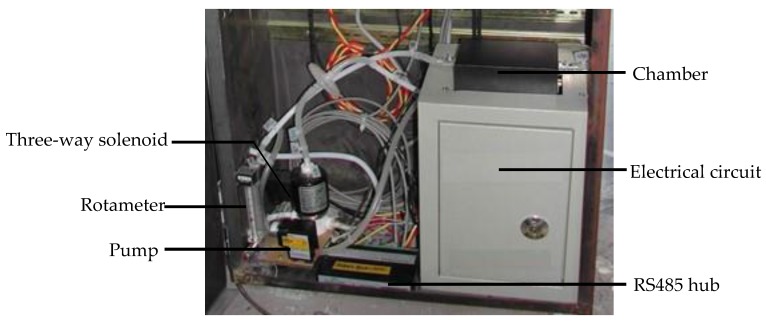
Electronic nose (E-nose) prototype.

**Figure 3:**
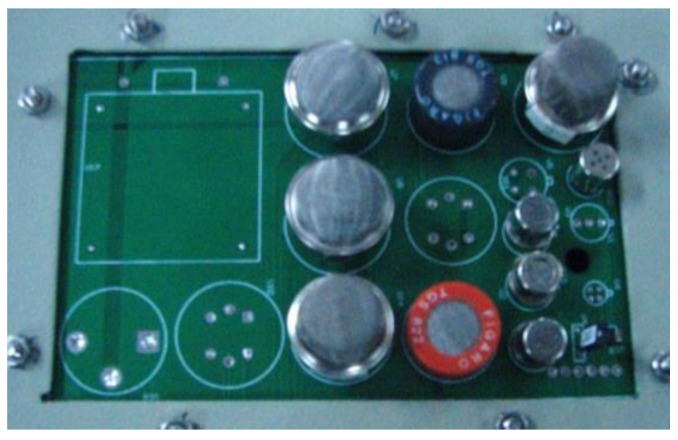
The sensors array in the chamber of E-nose.

The E-nose prototype (Figure 2) developed in this study was placed outside of the curing barn. The volume of the chamber in the E-nose is approximately 230 mL, and the sensors array (Figure 3) was located inside of the chamber. The inlet of the chamber was connected to one port of the three-way solenoid valves, which featured three port connections and two valve orifices, while the other two ports of the three-way solenoid valves were separately connected to the gas from the bulk curing barn and the ambient air that was purified by an air filter. The outlet of the chamber was connected to the pump. In the present study, the entire odor sampling period was one hour. In the first 15 min, a pump evacuated air from the chamber, and the valve of the pure air side was switched on. During this time, pure air entered the chamber from its inlet, and the gas sensors were expected to have no response. In the next 10 min, the valve of the pure air side switched off while the valve of the sampling gas turned on. The air in the bulk curing barn was evacuated into the chamber, and the gas sensors started to make responses to the volatile organic compounds (VOCs) released by the tobacco leaves during this period. After that, the sampling gas was blocked, and pure air was evacuated into the chamber again so as to purge the air in the chamber and to fully clean it. This process lasted another 15 min. In the rest of the time during the sampling period, the pump was switched off. The response curves of the gas sensors in one odor sampling period, also known as the original response curves, were similar to the static response curves [35]. When the pump was working, it evacuated gas from inside the chamber at the flow rate of 3 L/min. To maintain a relatively stable gas flux, a rotameter was installed between the pump and chamber. The on or off state of the pump, as well as the switching on or off of the three-way solenoid valves were controlled by the computer. The sensors array sent odor signals to the signal amplifier and regulation buffer. These analog signals then were converted to digital signals by a 12-bit ADC of the electrical circuit, at 3300 samples per second, and were sent to the computer. The computer processed the measured sensor signals, extracted the odor features, and analyzed them together with the other sensors’ data in order to achieve the intelligent control of the bulk tobacco curing schedule.

The design of the intelligent E-nose system included the selection of special sensors to measure the gas compounds in the curing barns. Based on previous extensive investigation on the existing odor measurement technologies and equipment, as well as on the various sensors available for measuring odor compounds, 10 gas sensors were selected, and they are listed in Table 1.

**Figure 4:**
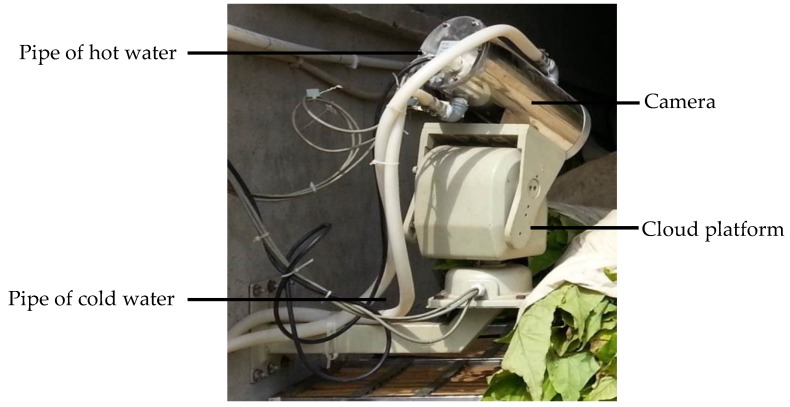
Image sampling set-up.

A machine vision system was developed in order to sense the image, specifically the color of the tobacco leaves during curing. The image sampling set-up is shown in Figure 4. A digital camera (CNB-ZCN-21Z22, CNB Technology Inc., Seoul, Korea) protected by a vacuum insulated shield (operating temperature range: −50~150 °C) with a cloud platform was fixed on the wall of the curing barns. The cloud platform made it possible to watch and monitor the tobacco leaves at any corner of the barns. Two water pipes were set around a camera and were used to cool down the camera, as a high temperature would be achieved during curing, which might affect the image quality of the camera. During the curing, images of the tobacco leaves were captured every hour, and were decoded by a video decoder card (DS4004HC, Hikvision Digital Technology Co., Ltd., Hangzhou, China) and sent to the computer, where the images were saved in bitmap format with the effective pixels number at 752 × 582. The image data were collected in such a way that the tobacco curing process could be protected and maintain its integrity.

The moisture data of the tobacco leaves were retrieved from a moisture meter (MC-7825P, Dalian Teren Industry Instruments Co., Ltd., Dalian, China). Two pin probes were inserted into the stem of the tobacco leaves to detect the water loss during curing. The moisture meter was connected to the computer with a RS-485 to RS-233 converter, and the moisture data were measured every hour during curing.

### 2.2. Data Sets

**Figure 5:**
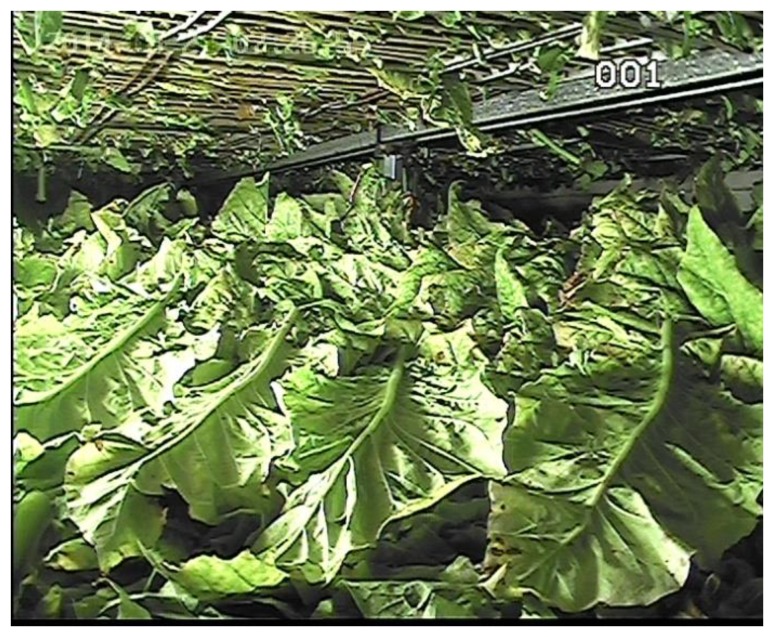
Inside of the curing barn loaded with tobacco.

The odor, image, and moisture data were collected through two test curing barns from July to September 2014, in Zunyi County, Guizhou Province, China. The tobacco leaves were cured following a three-stage curing theory. Each test barn (13,000 mm in length and 2600 mm in width) could be loaded with up to 5000 kg of tobacco leaves. All of the tobacco leaves were laid upon three layers of bamboo boards and were placed in a random way. The inside of the curing barn loaded with tobacco at the beginning of the curing process is illustrated in Figure 5. The data obtained from four experiments with good curing results were used in this research. Each experiment was a complete curing process. The available data sets are listed in Table 2. The period of each experiment lasts about one week. From the beginning to the end of the flue-curing process, the odor, image, and moisture data were collected every other hour by the ways described in Section 2.1. These real-time measurements might not affect or interrupt the tobacco curing process. The numbers of the measurement points varied a little for each experiment, and they were 157, 148, 147, and 151 respectively. Thus, a total of 603 measurement points were obtained. During data collecting, the curer’s adjustments in the tobacco curing schedule were recorded at the same time.

### 2.3. Controlled Parameters for Intelligent Bulk Tobacco Curing Schedule

**Figure 6:**
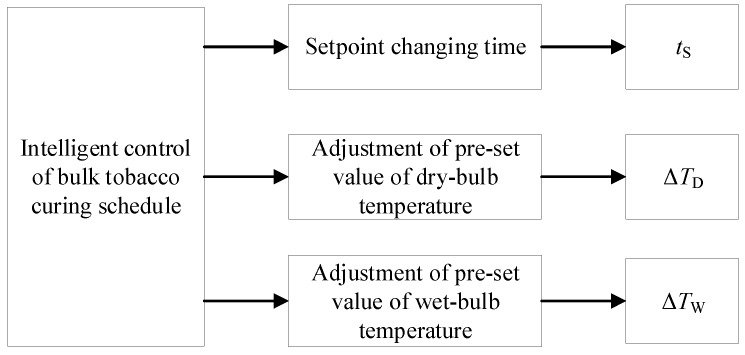
Controlled parameters for the intelligent control of a bulk tobacco curing schedule.

Analyzing the controlled parameters is the first step to model the intelligent control of a bulk tobacco curing schedule. The tobacco leaves were cured following a certain tobacco curing schedule. Before the curing began, a pre-set tobacco curing schedule was put in the memory of PLC. During the curing, the curing schedule could be adjusted by the curer as a result of the specific curing condition of the tobacco leaves. The intelligent control of the tobacco curing schedule means appropriately adjusting the controlled parameters of the curing schedule according to the information received from the multi-sensor data, which represent the image, odor, and moisture of the tobacco leaves. According to the curer’s operation of the curing schedule, the adjustment includes modifying the setpoint changing time ($t_{S}$), which denotes the curing time when the curing process changes from the current phase to the next; the adjustment of the pre-set value of the DBT, designated as $\Delta T_{D}$; and the adjustment of the pre-set value of WBT, designated as $\Delta T_{W}$. These controlled parameters are all shown in Figure 6.

**Figure 7:**
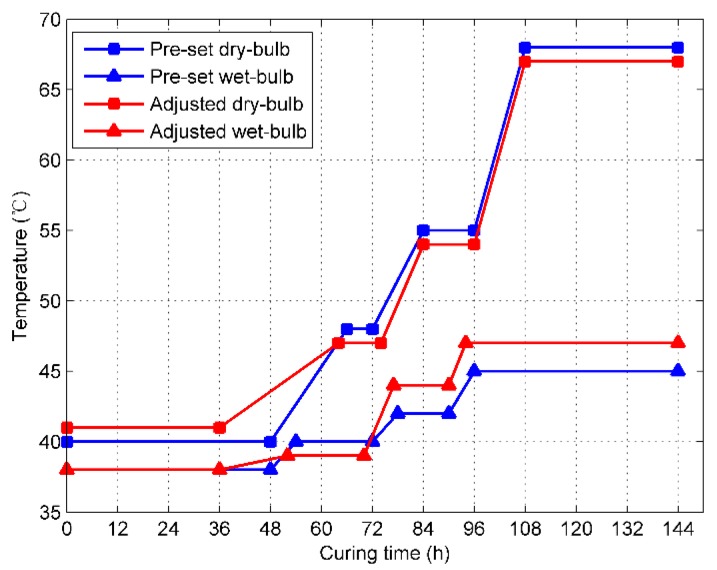
Pre-set and adjusted bulk tobacco curing schedules.

In order to give a detailed and clear description of the relationship between the controlled parameters and the tobacco curing schedule, an abridged general view of the tobacco curing schedule is plotted in Figure 7. The curing schedules in blue are the pre-set ones that are originally put in the memory of the PLC before curing, while the schedules in red are the adjusted ones that are made by the curer during curing. If the data is collected at the curing time of the 24th hour, the controlled parameters will be recorded as follows: $t_{S}$ is 36th hour, $\Delta T_{D}$ is 1 °C, and $\Delta T_{W}$ is 0 °C.

### 2.4. The Proposed Multi-Sensor Data Fusion Schemes for the Intelligent Control of the Tobacco Curing Schedule

Multi-sensor data fusion can provide a collaborative approach to improve prediction accuracy by using multiple sensors. In our study, there are the following four steps followed to accomplish multi-sensor data fusion:

Step 1: Acquire the versatile sensing information of the tobacco leaves during curing, including the odor, image, and moisture.

Step 2: Extract the multi-sensor data features of the odor, image, and moisture.

Step 3: Apply the proposed multi-sensor data fusion schemes to analyze the intelligent control of the tobacco curing schedule. 

Step 4: Evaluate the multi-sensor data fusion schemes by comparing their performance and prediction accuracy.

Three fusion schemes to analyze the intelligent control of the tobacco curing schedule were applied, including feature level fusion, decision level fusion, and hybrid fusion schemes.

**Figure 8:**
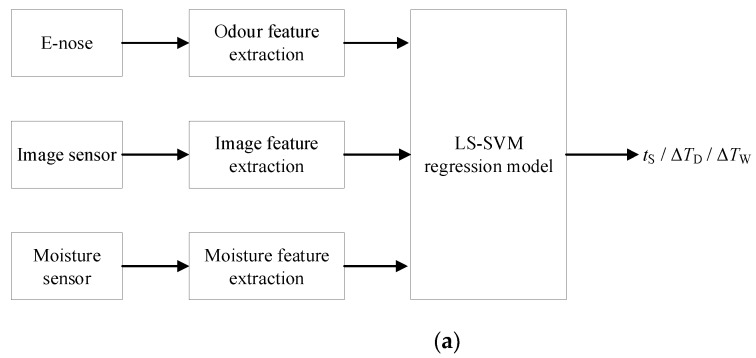

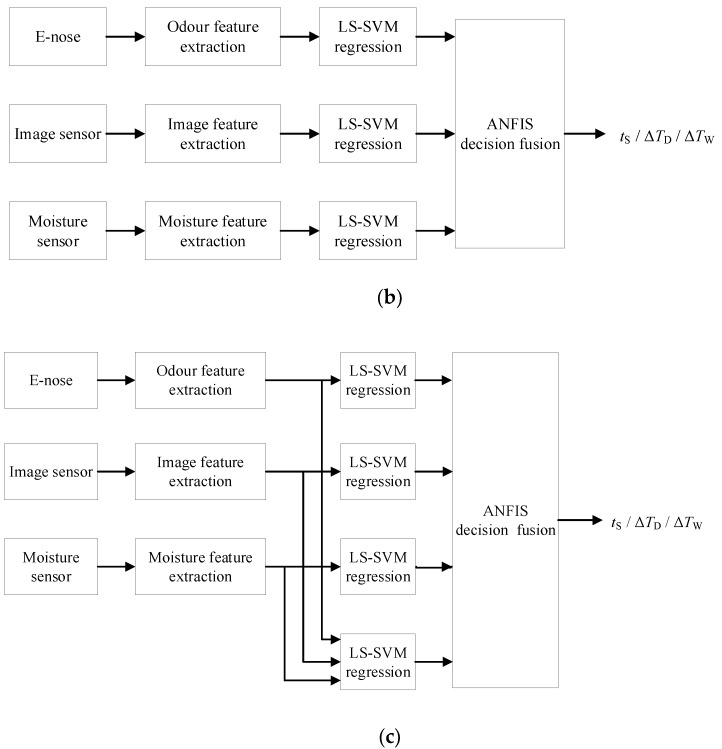
The proposed multi-sensor data fusion approaches are as follows: (**a**) feature level data fusion; (**b**) decision level data fusion; (**c**) hybrid multi-sensor data fusion. LS-SVM—least squares support vector machines; ANFIS—adaptive neuro-fuzzy inference system.

Figure 8 shows the multi-sensor data fusion schemes applied in our study. At the feature level fusion (Figure 8a), the multi-sensor data of the odor, image, and moisture were collected and pre-processed so as to extract the corresponding features of the tobacco leaves during curing. The least squares support vector machines (LS-SVM) regression model was chosen to analyze the intelligent control of the tobacco curing schedule, and its detailed definition is in Section 2.6. The inputs of the LS-SVM model are the joint features of odor, image, and moisture. The output is the controlled parameter of the bulk tobacco curing schedule.

The decision level fusion is shown in Figure 8b. The features of image, odor, and moisture were extracted and individually input into three different LS-SVM regression models for predicting the controlled parameter of the bulk tobacco curing schedule, which resulted in three sets of the output, all of which were then fed into an adaptive neuro-fuzzy inference system (ANFIS) in order to make the final decision of the adjustment. The ANFIS model will be defined in Section 2.7. The output of the ANFIS model is still the controlled parameter of the bulk tobacco curing schedule. 

The hybrid fusion scheme (Figure 8c) incorporates feature level fusion into decision level fusion. Four local decisions are made by four LS-SVM regression models, the inputs of which are the image, odor, moisture, and joint features, individually, which are then put into the ANFIS model to predict the final controlled parameter.

### 2.5. Feature Extraction Methods for Odor, Image, and Moisture

**Figure 9:**
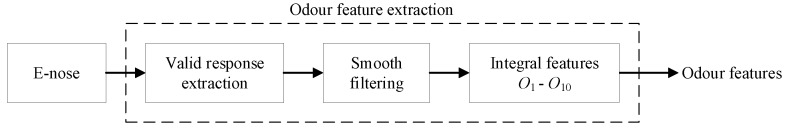
Odor features extraction method.

The odor features were extracted using the method presented in Figure 9. A valid response of the gas sensors should be extracted from the original response curves, as the noise and disturbance that appear when the sampling gas has not entered the chamber yet might impact the odor feature extraction. Then, after the smooth filtering of the valid response curves, this could be applied to extract the odor features. The methods of odor feature extraction that are normally used are based on the basic characteristics of the response curve [36,37,38]. In this study, integrals at a specific interval from the response curves were extracted. Integrals may represent the accumulative total of the reaction degree changing, which also reflects the gas sensor response equilibrium final steady state information, which is also used to distinguish between the different types and concentrations of odor. The integral of the response curve is given as follows:

(1) $$I_{t}=\int_{t_{1}}^{t_{2}}f\left(t\right)dt,$$

where f(t) is the transient response, $t_{1}$ is the time when the sampling air starts to enter the chamber, $t_{2}$ is the time when the sensors recovery completed, and *t* is the time index from $t_{1}$ to $t_{2}$. To simplify the calculation of Equation (1), the closed Newton–Cotes differential method [39] was used, and given as follows:

(2) $$I_{t}=\int_{t_{1}}^{t_{2}}f\left(t\right)dt\approx \frac{t_{2}-t_{1}}{6}\left[f\left(t_{1}\right)+4f\left(\frac{t_{1}+t_{2}}{2}\right)+f\left(t_{2}\right)\right].$$

Using Equation (2) to extract the integrals of the response curves, 10 entire odor features designated from *O*_1_ to *O*_10_ were extracted.

**Figure 10:**
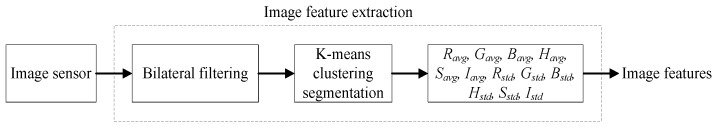
Image features extraction method.

During curing, images of the tobacco leaves were taken using the camera fixed on the wall of the curing barn, and were sent to the computer to process. The image feature extraction method (Figure 10) involved bilateral filtering and K-means clustering segmentation. The algorithms were described detailed by Wu et al. [9]. In total, 12 image features were extracted, which were noted as *R*_avg_, *G*_avg_, *B*_avg_, *H*_avg_, *S*_avg_, *I*_avg_, *R*_std_, *G*_std_, *B*_std_, *H*_std_, *S*_std_, and *I*_std_, where *R*_avg_, *G*_avg_, and *B*_avg_ are the mean values of the red component, the green component, and the blue component in the red, green, and blue (RGB) color model of the tobacco leaves image, respectively; *H*_avg_, *S*_avg_, and *I*_avg_ are the mean values of the hue component, the saturation component, and the brightness component in the hue, saturation, and intensity (HSI) color model of the tobacco leaves image, respectively; *R*_std_, *G*_std_, and *B*_std_ are the standard deviation of the red component, the green component, and the blue component in the RGB color model of the tobacco leaves image, respectively; and *H*_std_, *S*_std_, and *I*_std_ are the standard deviation of the hue component, the saturation component, and the brightness component in the HSI color model of tobacco leaves image, respectively.

**Figure 11:**
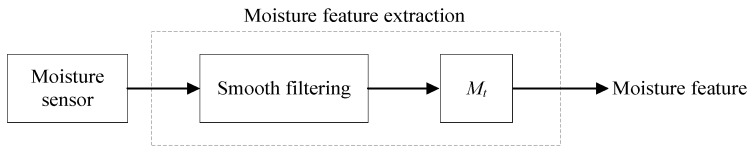
Moisture feature extraction method.

As for the moisture feature extraction (Figure 11), the data were collected from the moisture detector and then denoised using a smooth filter. The moisture feature labeled *M*_t_ was extracted.

### 2.6. LS-SVM Regression Model

The features of image, odor, and moisture were input into an LS-SVM model to analyze the local adjustment of the tobacco curing schedule. In this study, the LS-SVM regression model proposed by Suykens and Vandewalle [40] was applied.

Based on a training data set of *N* samples ${\left\{x_{i},y_{i}\right\}}_{i=1}^{N}$, where $x_{i}\in R^{p}$ represents a *p*–dimensional input data and $y_{i}\in R$ is the output variable that corresponds to $x_{i}$, the goal of the LS-SVM regression is to solve the minimization problem, which can be described as follows:

(3) $$\{\begin{array}{l} \underset{w,b,e}{\min}J\left(w,b,e\right)=\frac{1}{2}w^{T}w+\frac{\vartheta }{2}\sum_{i=1}^{N}e_{i}^{2}\;,\\ s.t.\;y_{i}=w^{T}\varphi \left(x_{i}\right)+b+e_{i\;},\;i=1,2,\ldots ,N, \end{array}$$

where $\varphi \left(\cdot \right):\;R^{p}\to R^{h}$ applies nonlinear mapping from the input space to a higher dimensional feature space, $w={\left[w_{1},w_{2},\ldots ,w_{h}\right]}^{T}$ is the weight vector, $e_{i\;}\in R$ is the error variance, *b* is a real constant, and ϑ is the trade-off (or penalty) parameter.

The Lagrangians of the optimization problem of Equation (3) can be formed as follows:

(4) $$ℒ\left(w,b,e:\alpha \right)=J\left(w,b,e\right)-\sum_{i=1}^{N}\alpha_{i}\left\{w^{T}\varphi \left(x_{i}\right)+b+e_{i}-y_{i}\right\},$$

where $\alpha_{i}\in R$ is the Lagrange multiplier. The conditions for optimality are the following [41]:

(5) $$\{\begin{array}{l} \frac{\partial ℒ}{\partial w}=0\to w=\sum_{i=1}^{N}\alpha_{i}\varphi \left(x_{i}\right),\\ \frac{\partial ℒ}{\partial b}=0\to \sum_{i=1}^{N}\alpha_{i}=0,\\ \frac{\partial ℒ}{\partial e_{i}}=0\to \alpha_{i}=\vartheta e_{i},\;i=1,2,\ldots ,N,\;\\ \frac{\partial ℒ}{\partial \alpha_{i}}=0\to w^{T}\varphi \left(x_{i}\right)+b+e_{i}-y_{i}=0,\;i=1,2,\ldots ,N. \end{array}$$

After the elimination of w and e, the output y(x) corresponding to a new input vector **x** can be obtained in the form of the following:

(6) $$y\left(x\right)=\sum_{i=1}^{N}\alpha_{i}K\left(x,x_{i}\right)+b,$$

where $\alpha_{i}$ and b can be solved by Equation (5), and $K\left(x,x_{i}\right)=\varphi {\left(x\right)}^{T}\;\varphi \left(x_{i}\right)$ is the kernel function of the LS-SVM model. In this study, radial basis function (RBF) kernel was chosen, which is given as follows:

(7) $$K\left(x,x_{i}\right)=\exp\left\{-\frac{{||x-x_{i}||}^{2}}{\sigma^{2}}\right\},\;i=1,2,\ldots ,N,$$

where σ is a constant representing the “width” of this Gaussian function.

### 2.7. ANFIS Decision Model

At the decision level fusion and hybrid fusion, ANFIS was applied to make the final decision on the adjustments of the tobacco curing schedule. The inputs of the ANFIS are the local decisions made by the LS-SVM models. A Sugeno-type fuzzy relation was used in this study, and the linguistic labels of each input are small, medium, and large. The membership function applied is triangular-shaped membership function, given as follows:

(8) $$\mu_{A_{ij}}\left(x_{i}\right)=\{\begin{array}{cc} 0, & x_{i}\leq \delta_{ij}\\ \frac{x_{i}-\delta_{ij}}{\theta_{ij}-\delta_{ij}}, & \delta_{ij}\leq x_{i}\leq \;\theta_{ij}\\ \frac{\rho_{ij}-x_{i}}{\rho_{ij}-\theta_{ij}}, & \theta_{ij}\leq x_{i}\leq \;\rho_{ij}\;\\ 0, & \rho_{ij}\leq x_{i} \end{array}\;i=1,\;2,\ldots ,\;M;\;j=1,\;2,\;3;$$

where $x_{i}$ is the *i*-th input linguistic variable, *M* is the number of the input linguistic variables (for decision level fusion, *M* is 3, and for hybrid fusion, *M* is 4), $A_{ij}$ is the *j*-th linguistic label associated with $x_{i}$, and $\delta_{ij}$, and $\theta_{ij}$, and $\rho_{ij}$ are the parameters of the triangular-shaped function.

There are five layers in the ANFIS model. They are the fuzzification layer, rule operation layer, normalization layer, consequent layer, and aggregation layer, sequentially. The output of the ANFIS model is expressed as follows:

(9) $$F=\sum_{k=1}^{3^{M}}\bar{w_{k}}h_{k}=\frac{\sum_{k}w_{k}h_{k}}{\sum_{k}w_{k}}\;\;\;k=1,\;2,\ldots ,\;3^{M},$$

where $w_{k}$ is the firing strength of the *k*-th rule, given as follows:

(10) $$w_{k}=\prod_{i=1}^{M}\mu_{A_{ij_{i}}}\left(x_{i}\right)\;\;\;\;i=1,\;2,\;\ldots ,M;\;j_{i}=1,\;2,\;3;\;k=1,\;2,\ldots ,\;3^{M},$$

and $h_{k}$ is the consequential output of the *k*-th rule, which takes the following form:

Rule 1: IF $x_{1}$ is $A_{11}$ and $x_{2}$ is $A_{21}$ … and $x_{M}$ is $A_{M1}$,
Then $h_{1}=a_{0}^{1}+a_{1}^{1}x_{1}+a_{2}^{1}x_{2}+\ldots +a_{M}^{1}x_{M}$
Rule 2: IF $x_{1}$ is $A_{11}$ and $x_{2}$ is $A_{21}$ … and $x_{M}$ is $A_{M2}$,
Then $h_{2}=a_{0}^{2}+a_{1}^{2}x_{1}+a_{2}^{2}x_{2}+\ldots +a_{M}^{2}x_{M}$
…
Rule *k*: IF $x_{1}$ is $A_{1j_{1}}$ and $x_{2}$ is $A_{2j_{2}}$ … and $x_{i}$ is $A_{ij_{i}}$ … and $x_{M}$ is $A_{Mj_{M}}$,
Then $h_{k}=a_{0}^{k}+a_{1}^{k}x_{1}+a_{2}^{k}x_{2}+\ldots +a_{i}^{k}x_{i}+\ldots +a_{M}^{k}x_{M}$,

where $a_{i}^{k}$ is the Sugeno parameter,$\;i=1,\;2,\;\ldots ,M;\;j_{i}=1,\;2,\;3;\;k=1,\;2,\ldots ,\;3^{M}$.

### 2.8. Evaluation of the Performance of the Data Fusion Schemes

To assess the performance of the multi-sensor data fusion schemes, the evaluation parameters of the coefficient of determination (R^2^), which is the square of correlation coefficient; root mean square error (RMSE); and mean absolute error (MAE) are used.

## 3. Results and Discussions

### 3.1. Odour Data Pre-Processing Analysis 

**Figure 12:**
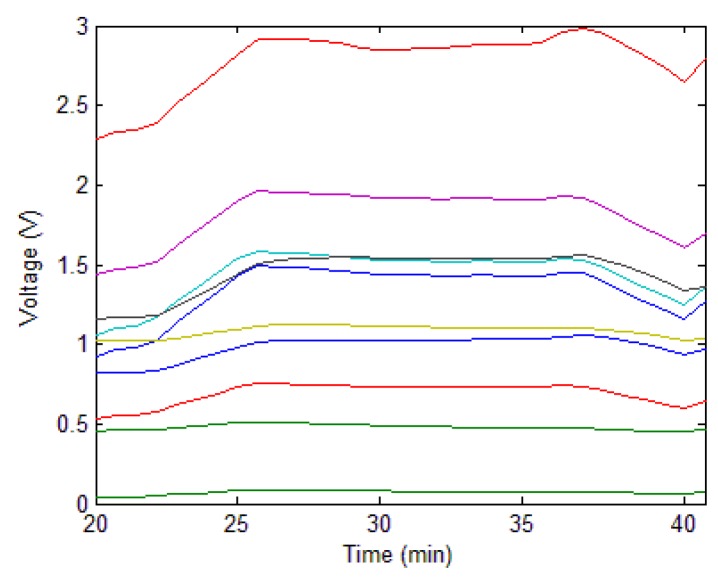
Pre-processing result of the E-nose response data to the air in the bulk curing barn.

The pre-processing of odor data was applied. The data were cropped and smoothed, and the results are illustrated in Figure 12.

### 3.2. Odor, Image, and Moisture Features of the Tobacco Leaves during Curing

**Figure 13:**
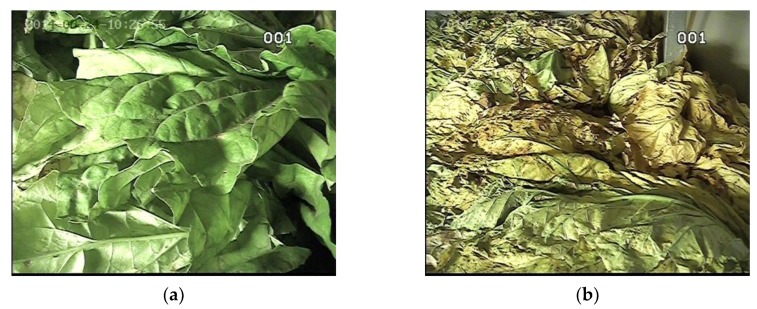
The raw images of tobacco leaves at the leaf-yellowing stage in the different curing phases: (**a**) early phase; (**b**) middle phase.

**Figure 14:**
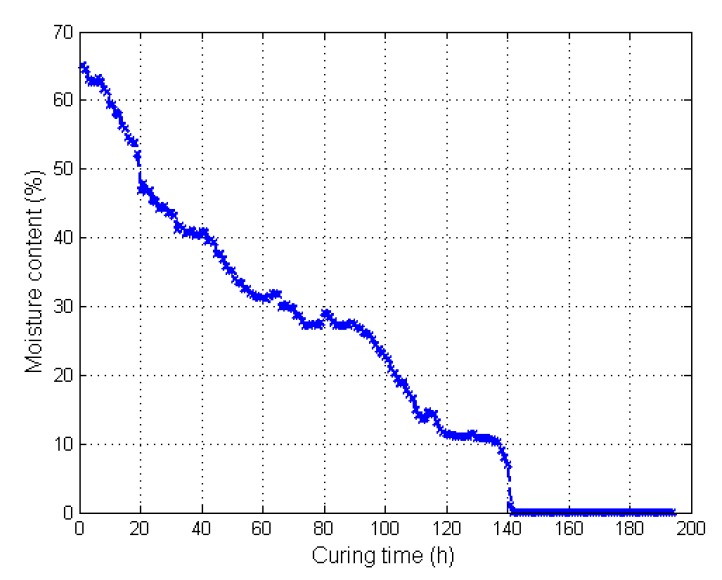
The moisture content of tobacco during curing.

**Figure 15:**
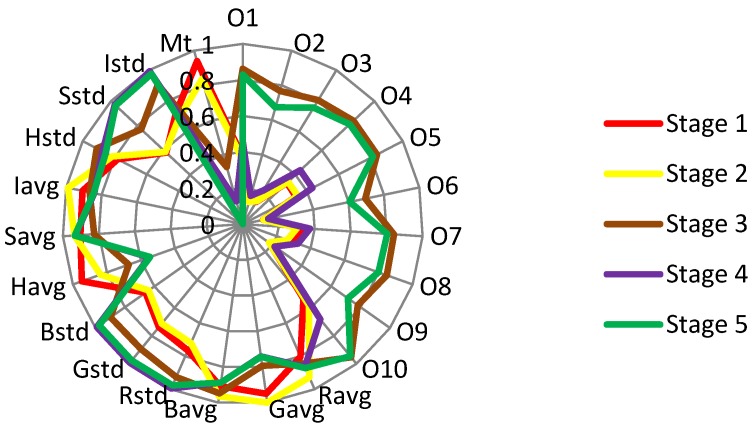
Radar chart of the odor, image, and moisture features in five stages.

The odor, image, and moisture data, which were collected by the E-nose, image sensor, and moisture sensor, respectively, were processed by the methods described in Section 2.5. The raw images of the tobacco leaves at the leaf-yellowing stage are shown in Figure 13, and the moisture content of the tobacco during curing is illustrated in Figure 14. The features extracted in the five different stages during tobacco curing are illustrated in the radar chart of Figure 15. The data from stage 1 to stage 5 were collected at the beginning of the curing, leaf-yellowing stage, leaf-drying stage, stem-drying stage, and the end of the curing, correspondingly. The feature vectors were normalized to unit length so as to be appropriately shown in the radar chart.

At the early period of curing (stages 1 and 2), the odor features changed a little from the beginning to the leaf-yellowing stage. Meanwhile, the strengths of the odor in this period were the weakest in five stages, which means that the fragrance and some other VOCs of the tobacco leaves had not been released yet at the leaf-yellowing stage. The image features from this period changed a lot, especially for the features of *R*_avg_ and *H*_avg_. Also, the moisture of the tobacco leaves decreased from the very beginning to the leaf-yellowing stage.

At the leaf-drying stage (stage 3), the odor features made the most significant change and became the strongest out of the whole curing, indicating that the fragrance and VOCs come out largely at this stage, which is a similar conclusion to Song et al. [42]. The image features at this period continued to make changes, and the color of the tobacco leaves curing at this stage tended to be fixed. The moisture feature decreased a lot, and the decreasing amplitude was much larger than that from stage 1 to stage 2.

At the stem-drying stage (stage 4), the odor strength decreased, the image features were made smaller, and the moisture content of the tobacco leaves continued to decrease.

At the end of the curing (stage 5), the odor strength became strong again, the image features had nearly no change compared with that at the stem-drying stage, and the moisture content became nearly 0, indicating that the tobacco leaves were cured to be almost dry. The tobacco leaves were cured to be completely dry after curing [43].

### 3.3. Performance Analysis of the Proposed Multi-Sensor Data Fusion Schemes

In total, 603 measurement points were obtained from four experiments, of which 25% (151) were randomly selected for testing and 75% (452) were used for training. For the LS-SVM model, the optimization routine and cost function were set as grid search and cross validation, respectively. For the ANFIS model, the max iteration was 1000, the step size was initialized to be 0.05, and all of the consequent parameters were set to be 0 initially.

**Figure 16:**
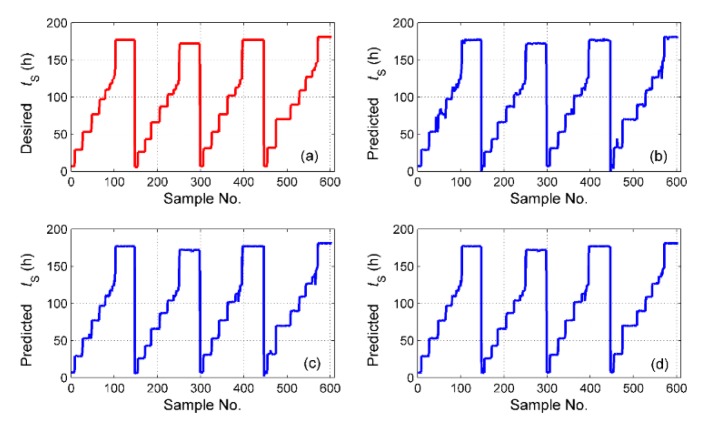
The simulation results of $t_{s}$ for different fusion schemes, namely: (**a**) desired $t_{s}$; (**b**) predicted $t_{s}$ for feature level data fusion; (**c**) predicted $t_{s}$ for decision level data fusion; (**d**) predicted $t_{s}$ for hybrid multi-sensor data fusion.

**Figure 17:**
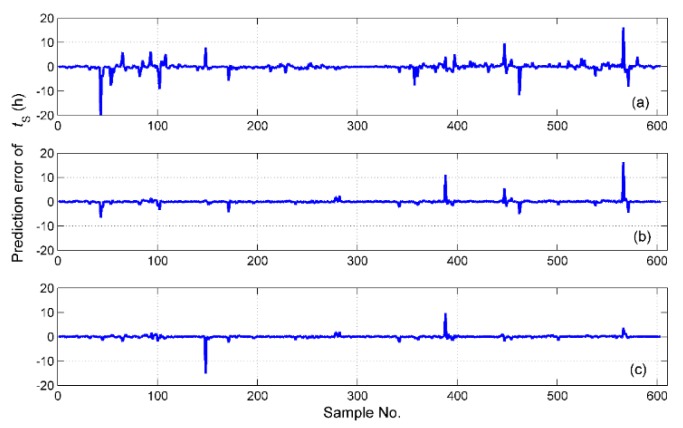
The prediction errors of $t_{s}$ for the different fusion schemes, namely: (**a**) feature level data fusion; (**b**) decision level data fusion; (**c**) hybrid multi-sensor data fusion.

**Figure 18:**
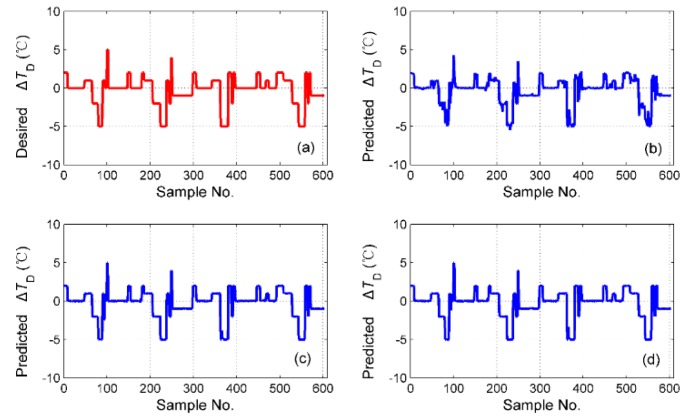
The simulation results of $\Delta T_{D}$ for different fusion schemes, namely: (**a**) desired $\Delta T_{D}$; (**b**) predicted $\Delta T_{D}$ for feature level data fusion; (**c**) predicted $\Delta T_{D}$ for decision level data fusion; (**d**) predicted $\Delta T_{D}$ for hybrid multi-sensor data fusion.

**Figure 19:**
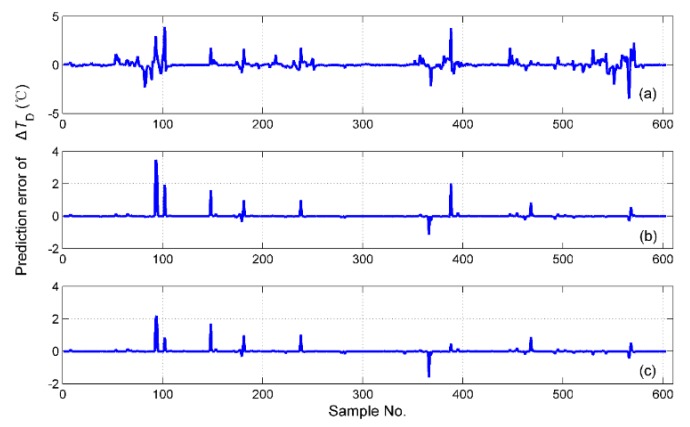
The prediction errors of $\Delta T_{D}$ for different fusion schemes, namely: (**a**) feature level data fusion; (**b**) decision level data fusion; (**c**) hybrid multi-sensor data fusion.

**Figure 20:**
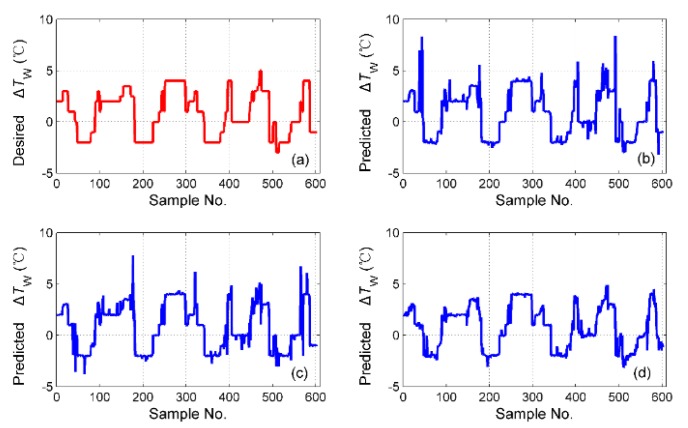
The simulation results of $\Delta T_{W}$ for different fusion schemes, namely: (**a**) desired $\Delta T_{W}$; (**b**) predicted $\Delta T_{W}$ for feature level data fusion; (**c**) predicted $\Delta T_{W}$ for decision level data fusion; (**d**) predicted $\Delta T_{W}$ for hybrid multi-sensor data fusion.

**Figure 21:**
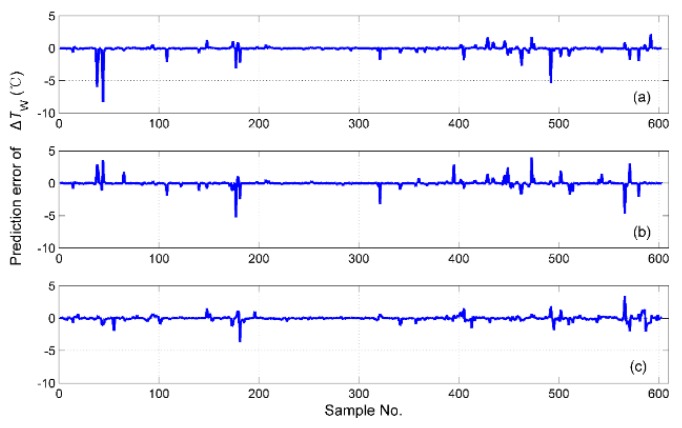
The prediction errors of $\Delta T_{W}$ for different fusion schemes, namely: (**a**) feature level data fusion; (**b**) decision level data fusion; (**c**) hybrid multi-sensor data fusion.

The simulation results of $t_{S}$ for the different fusion schemes are shown in Figure 16, and the corresponding prediction error is shown in Figure 17. It is obvious that the best performance of the prediction is the hybrid fusion model, for which the prediction results are shown in Figure 16d and Figure 17c. The feature level fusion scheme provides relatively poor predictions (Figure 16b and Figure 17a), in which the maximum error achieved 20 h, which is unacceptable for curing hours. The simulation results of $\Delta T_{D}$ are shown in Figure 18, and the prediction error is shown in Figure 19. The prediction results of the feature level fusion (Figure 18b) do not agree with the desired results (Figure 18a). The simulation results of the decision level fusion and hybrid fusion are quite similar. The simulation results of $\Delta T_{W}$ are shown in Figure 20, and the prediction error is shown in Figure 21. Both of the results made by the feature level (Figure 20b) and decision level (Figure 20c) fusions are not satisfied. Apparently, the hybrid fusion scheme (Figure 20d) makes the best performance among the three data fusion schemes.

### 3.4. Statistical Comparison

To make a more specific comparison of the different fusion schemes, the detailed statistical parameters of the test results are listed in Table 3. For the statistical parameters of R^2^, RMSE, and MAE, the best performance was made by the hybrid fusion scheme. For a better understanding of the good performance of the hybrid fusion scheme, *P_S1_* (percentage of $t_{S}$ data with an absolute error smaller than 1 h), *P_D1_* (percentage of $\Delta T_{D}$ data with an absolute error smaller than 0.5 °C), and *P_W1_* (percentage of $\Delta T_{W}$ data with an absolute error smaller than 0.5 °C) are also given in the table. According to practical experience, the higher these percentages, the smaller the effect of these errors on tobacco curing. Tested by the hybrid fusion scheme, *P_S1_* was 7.3% beyond the decision level fusion scheme and 40.6% beyond the feature level fusion scheme, while for *P_D1_*, an improvement of 5.2% was recorded over the decision level fusion scheme and a gain of 19.8% was achieved over the feature level fusion scheme. Similarly, the *P_W1_* tested by hybrid fusion was 12.2% above the decision level fusion and 16% above the feature level fusion. In Table 3, *P_S2_* (percentages of $t_{S}$ data with absolute error larger than 5 h), *P_D2_* (percentage of $\Delta T_{D}$ data with absolute error larger than 1 °C), and *P_W2_* (percentage of $\Delta T_{W}$ data with absolute error larger than 1 °C) were also recorded. The higher the values of *P_S2_*, *P_D2_*, and *P_W2_*, the greater the effect of the error on tobacco curing. The results demonstrate that the prediction performance of the proposed hybrid multi-sensor data fusion is superior to those obtained by the other two data fusion schemes.

In addition, we have compared our proposed hybrid fusion scheme with that of Zhang et al. [24]. They used a three-layer neural network trained by a typical back propagation learning algorithm in a feature level in order to provide the local decision by each kind of the tobacco features and applied a weighted sum model (WSM) to make the global decision. Tested by the above method, the coefficient of determination R^2^ for the controlled parameters of $t_{S}$, $\Delta T_{D}$, and $\Delta T_{W}$ are 0.9636, 0.8472, and 0.7945, decreasing by 0.0355, 0.1117, and 0.1534, respectively. For $t_{S}$, there is 15.2% of data with an absolute error larger than five hours, 13.9% bigger than that obtained by the hybrid fusion scheme, which might result in an unsatisfactory curing control process, especially when the error occurs at the leaf-yellowing stage. For the WSM in Zhang et al. [24], the simple multi-criteria decision analysis method is used, and the weight of each local decision is determined by the relative importance of each characteristic of the tobacco leaves, that is, odor, color, and moisture, which highly depends on the empirical analysis.

One could tell from the experiment results that the intelligent control of the curing process is promising in our system. The predicted results for $\Delta T_{W}$ still need to be promoted. For commercial use, the future work of this study should focus on the optimization of the odor data processing to improve the effectiveness of the odor feature extraction and selection, so that a more accurate control process might be achieved.

## 4. Conclusions

This study put forward the controlled parameters of the bulk tobacco curing schedule, namely, the setpoint changing time labeled $t_{S}$, the adjustment of the pre-set value of the DBT labeled $\Delta T_{D}$, and the adjustment of the pre-set value of WBT labeled $\Delta T_{W}$, which is significant and important for the construction of an intelligent bulk tobacco curing system. To fully imitate the curer’s operation of the adjustments on the tobacco curing schedule, three types of sensors were applied, that is, a gas sensor, image sensor, and moisture sensor. The odor, image, and moisture features extraction methods, as well as the way to extract the valid response curves of the gas sensors, were presented. Multi-sensor data fusion schemes were proposed to analyze the intelligent control of the bulk tobacco curing schedule.

Three multi-sensor data fusion schemes were applied, including feature level fusion, decision level fusion, and hybrid fusion schemes. The prediction results demonstrate that a hybrid fusion scheme that incorporated the feature level fusion into the decision level performed best. The coefficient of determination R^2^ for $t_{S}$ is 0.9991, 0.9589 for $\Delta T_{D}$, and 0.9479 for $\Delta T_{W}$. There were 91.3% of testing data with an absolute error less than 1 h for $t_{S}$, 94.8% of data with an absolute error less than 0.5 °C for $\Delta T_{D}$, and 86.7% of the data with an absolute error less than 0.5 °C for $\Delta T_{D}$. The high prediction accuracy that the proposed hybrid fusion scheme achieved made it a feasible, reliable, and effective method for the intelligent control of the tobacco curing schedule.

## Figures and Tables

**Table 1:** Sensors used for the developed electronic nose (E-nose).

Name of Sensor	Compounds to be Detected	Material
TGS 2600	Hydrogen and carbon monoxide.	Metal oxide
TGS 2602	Ammonia, H_2_S, and toluene.	Metal oxide
TGS813	Methane, propane, and butane.	Tin dioxide
TGS822	Carbon monoxide, methane, combustible gases, and vapors of organic solvents.	Tin dioxide
TGS825	Hydrogen sulfide.	Tin dioxide
TGS826	Iso-butane, hydrogen, ammonia, and ethanol.	Metal oxide
WSP2111	Toluene, benzene, alcohol, and acetone.	Metal oxide
MQ135	NH_3_, NOx, alcohol, benzene, smoke, and CO_2_.	Tin dioxide
MQ138	Benzene, n-Hexane, NH_3_, alcohol, smoke, and CO.	Tin dioxide
SP3S-AQ2	Methane, iso-butane, CO, hydrogen, and ethanol.	Tin dioxide

**Table 2:** Data sets.

Experiment No.	Experimental Period (day/month)	Number of Measurement Points	Test Curing Barn	Tobacco Stalk Position	Type of Tobacco
B146705	24/08–01/09	157	No. 2	Upper	GY2
B146704	14/08–21/08	148	No. 2	Upper	GY2
B146604	14/08–21/08	147	No. 1	Upper	NJ3
B146605	24/08–01/09	151	No. 1	Upper	GY2
Total number of measurement points		603	----	----	----

**Table 3:** The performance comparison of different fusion schemes on intelligent control of tobacco curing schedule. RMSE—root mean square error; MAE—mean absolute error; R^2^—coefficient of determination.

Fusion Schemes	Statistical Parameters	Adjustment of the Tobacco Curing Schedule		
		*t_S_* (h)	Δ*T_D_* (°C)	Δ*T_W_* (°C)
Feature level fusion	R^2^	0.9962	0.7552	0.7826
	RMSE	3.4184	0.8715	1.2105
	MAE	1.9085	0.4731	0.5631
	*P_S1_/P_D1_/P_W1_* (%)	50.7	75	70.7
	*P_S2_/P_D2_/P_W2_* (%)	12.7	15.3	16
Decision level fusion	R^2^	0.9987	0.9183	0.8049
	RMSE	2.0279	0.5056	1.1054
	MAE	0.8550	0.1560	0.5917
	*P_S1_/P_D1_/P_W1_* (%)	84	89.6	74.5
	*P_S2_/P_D2_/P_W2_* (%)	4.7	4.9	12.7
Hybrid fusion	R^2^	0.9991	0.9589	0.9479
	RMSE	1.6521	0.3595	0.5362
	MAE	0.6428	0.1268	0.4769
	*P_S1_/P_D1_/P_W1_* (%)	91.3	94.8	86.7
	*P_S2_/P_D2_/P_W2_* (%)	1.3	3.3	9.1
